# Cerebello-Cortical Alterations Linked to Cognitive and Social Problems in Patients With Spastic Paraplegia Type 7: A Preliminary Study

**DOI:** 10.3389/fneur.2020.00082

**Published:** 2020-02-25

**Authors:** Michela Lupo, Giusy Olivito, Silvia Clausi, Libera Siciliano, Vittorio Riso, Marco Bozzali, Filippo M. Santorelli, Gabriella Silvestri, Maria Leggio

**Affiliations:** ^1^Ataxia Laboratory, IRCCS Fondazione Santa Lucia, Rome, Italy; ^2^Department of Psychology, Sapienza University of Rome, Rome, Italy; ^3^IRCCS Fondazione Santa Lucia, Rome, Italy; ^4^Department of Neurology, IRCCS Fondazione Policlinico Agostino Gemelli, Rome, Italy; ^5^Clinical Imaging Science Center, Brighton and Sussex Medical School, Brighton, United Kingdom; ^6^Molecular Medicine, IRCCS Fondazione Stella Maris, Pisa, Italy

**Keywords:** spastic paraplegia type 7, cerebellum, cognition, social skills, CCAS, functional connectivity, voxel-based morphometry

## Abstract

Spastic paraplegia type 7 (SPG7), which represents one of the most common forms of autosomal recessive spastic paraplegia (MIM#607259), often manifests with a complicated phenotype, characterized by progressive spastic ataxia with evidence of cerebellar atrophy on brain MRI. Recent studies have documented the presence of peculiar dentate nucleus hyperintensities on T2-weighted images and frontal executive dysfunction in neuropsychological tests in SPG7 patients. Therefore, we decided to assess whether any particular MRI pattern might be specifically associated with SPG7 mutations and possibly correlated with patients' cognitive profiles. For this purpose, we evaluated six SPG7 patients, studying the cerebello-cortical network by MRI voxel-based morphometry and functional connectivity techniques, compared to 30 healthy control subjects. In parallel, we investigated the cognitive and social functioning of the SPG7 patients. Our results document specific cognitive alterations in language, verbal memory, and executive function in addition to an impairment of social task and emotional functions. The MRI scans showed a diffuse symmetric reduction in the cerebellar gray matter of the right lobule V, right Crus I, and bilateral lobule VI, together with a cerebral gray matter reduction in the lingual gyrus, precuneus, thalamus, and superior frontal gyrus. The evidence of an over-connectivity pattern between both the right and left cerebellar dentate nuclei and specific cerebral regions (the lateral occipital cortex, precuneus, left supramarginal gyrus, and left superior parietal lobule) confirms the presence of cerebello-cortical dysregulation in different networks involved in cognition and social functioning in SPG7 patients.

## Introduction

Mutations in the spastic paraplegia type 7 (SPG7) gene, encoding the mitochondrial protein paraplegin (MIM^*^602783), are responsible for a rare form of hereditary spastic paraplegia (1, 2) and are usually transmitted as an autosomal recessive trait, which can cause both pure and complex phenotypes (1). The most common clinical presentation of SPG7 is spastic-ataxia, which is often associated with optic atrophy, ptosis, and ophthalmoparesis, and brain MRI usually shows the presence of mild or moderate cerebellar atrophy (3–6).

Despite the fact that cerebellar degeneration is a hallmark of the SPG7 complex phenotype, to date, only a few studies have attempted to characterize the pattern of cerebellar involvement in correlation with other brain structures. In this regard, Hewamadduma et al. (7) proposed cerebellar vermis atrophy associated with an increase in the T2 signal on MRI images of the dentate nucleus (DN) as a possible specific MRI pattern in SPG7 (7). The DN alteration is of particular interest, given the cerebellar anatomy. Indeed, the DN is the major cerebellar output channel connecting to the cerebral cortex (8), and modifications in functional connectivity (FC) within specific cerebello-cortical networks have already been described in patients affected by other forms of cerebellar atrophy (9–12) and linked to motor, cognitive, and behavioral symptoms (13–22).

In addition to the recognized role in motor functions, many research studies have assessed an important role for the cerebellum in cognition, with a related specific cognitive pattern known as cerebellar cognitive affective syndrome (CCAS) or Schmahmann's syndrome. Interestingly, in addition to spastic ataxia, a peculiar cognitive/behavioral phenotype characterized by “emotional disconnection” has recently been described in an SPG7 patient (23).

On the basis of this evidence, we set up a preliminary study on the structural and functional cerebellar networks of six SPG7 patients to identify a potential MRI pattern underlying clinical alterations in specific cognitive and affective functions.

## Materials and Methods

### Participants

Six adult male patients (mean age/SD = 46.33/12.93) with spastic ataxia and diagnosis of SPG7 were recruited for this study by the Neurology Department of Fondazione Policlinico Gemelli, IRCCS. All of them showed cerebellar atrophy on their diagnostic brain MRI. The study protocol, including MRI acquisition and clinical and cognitive assessment of patients, was performed at the IRCCS Fondazione Santa Lucia. Confirmation of the presence of macroscopic cerebellar alterations on T2-weighted brain MRI scans was given by an expert neuro-radiologist and represented a major inclusion criterion. At the time of enrolment, none of the SPG7 patients presented with any current or past diagnosis of other neurological/psychiatric disorders. Cerebellar motor deficits were assessed using the International Cooperative Ataxia Rating Scale (ICARS) (24), which has a global score ranging from 0 (absence of any motor deficits) to 100 (presence of motor deficits at the highest degree). The patients' genetic, clinic, and demographic data are reported in Table 1. For the MRI analysis, 30 healthy subjects (HS), matched for age and sex (mean age /SD = 38.43/13.64; M/F = 30/0), were recruited from the IRCCS Fondazione Santa Lucia as a control group. For the HS group, conventional MRI was inspected to exclude any pathological conditions according to the inclusion criteria.

**Table 1:** Demographic, molecular, and clinical characteristics of SPG7 patients.

**Group**	**Age**	**Age at onset**	**Mutation**	**Protein**	**ICARS**
CB_1	54	25	c.1779+1G>T + c.184_286del	Splice site + Exon 2 deletion	56
CB_2	53	45	c.1450-1del]_[c.1450_1457del] + c.1931C>A	Splice site + p.Thr644Asn	27
CB_3	55	45	c.637 C>T/–	p.Arg213*/–	9
CB_4	54	43	c.637C>T + c.1529C>T	p.Arg213*+ p.Ala510Val	35
CB_5	23	C	c.1013G>T/–	p.Gly338Val/-	17
CB_6	39	A	c.1369C>T + c.1617delC	p. Arg457*+ p.Val540CfsX52	7
Mean (sd)	46.33 (12.93)	35.33 (10.86)	–	–	25.17 (18.49)

*SD, standard deviations; C, childhood; A, adolescence*.

The experimental protocol, designed according to the Helsinki Declaration, was approved by the Ethics Committee of the IRCCS Fondazione Santa Lucia. Written, informed consent was obtained from each subject per the Helsinki Declaration.

### Behavioral Assessments

All patients underwent neuropsychological evaluation to investigate their cognitive profiles and social cognition abilities. Moreover, the Schmahmann's syndrome scale (SSS) was used (25) to evaluate the presence of cerebellar cognitive affective syndrome CCAS (15).

The neuropsychological assessment included the following tasks:

**Intellectual level**: Wechsler Adult Intelligence Scale (WAIS-IV) (26); Raven's Progressive Matrices '47 test (27).
**Verbal Memory**: immediate and delayed recall of prose memory (28); short- and long-term Rey's 15 words test (29); forward and backward digit span (30, 31).
**Visuospatial Memory**: Rey-Osterrieth Complex Figure Test (recall) (28); forward and backward Corsi (32).
**Visuospatial abilities**: Rey-Osterrieth Complex Figure Test (copy) (28).
**Language**: naming objects, naming verbs, and naming objects described by the examiner (33); generation of sentences (34).
**Executive Functions**: Stroop Test (“time effect” and “error effect”) (35); phonological fluency (36); verbal fluency (37); Wisconsin Card Sorting Test (WCST) (number of errors and perseverative errors) (38); Tower of London procedure (TOL) (39).
**Attention**: Multiple Features Target Cancellation task (40), and the Trail Making Test B-A (TMT B-A) (41).

To evaluate social cognition abilities, patients underwent the following tasks (see Appendix in Supplementary Material for details):

**The Reading the Mind in the Eyes test** (RME) (42, 43) was used to assess the first stage (automatic) of attributing relevant mental states to others regardless of the context.
**The Emotion Attribution test** (EA) (44, 45) was used to assess the ability to attribute emotions to others in a social context.
**The Theory of Mind** (ToM) task (44–47) was used to assess the more advanced concepts of the ToM, such as double bluff, white lies, and persuasion.

### MRI Acquisition Protocol

All patients and HSs underwent MRI examination at 3 T (Magnetom Allegra, Siemens, Erlangen, Germany) that included the following acquisitions: (1) dual-echo turbo spin-echo [TSE] (TR = 6,190 ms, TE = 12/109 ms); (2) fast-FLAIR (TR = 8,170 ms, 204TE = 96 ms, TI = 2,100 ms); (3) T1-weighted 3D high-resolution scan (3D modified driven equilibrium Fourier transform (MDEFT) (TR = 1,338 ms, TE = 2.4 ms, matrix = 256 × 224 × 176, in-plane FOV = 250 × 250 mm^2^, slice thickness = 1 mm); (4) T2^*^-weighted echo-planar imaging (EPI) sensitized to blood oxygenation-level dependent imaging (BOLD) contrast (TR = 2,080 ms, TE = 30 ms, 32 axial slices parallel to the AC-PC line, matrix 64 × 64, pixel size 3 × 3 mm^2^, slice thickness 2.5 mm, flip angle 70°) for resting-state fMRI. BOLD echo-planar images were collected during rest for a 7 min and 20 s period, resulting in a total of 220 volumes. During this acquisition, subjects were instructed to keep their eyes closed, not to think of anything in particular, and not to fall asleep.

### Statistical Analysis

#### Behavioral Assessments

To evaluate the general neuropsychological and social cognition profiles, the raw scores obtained in each task were corrected for age and educational values according to the corresponding published normative data.

#### Image Processing and Data Analysis

An independent two-sample *T*-test ensured that SPG7 patients and HS did not differ in terms of age (*t* = −1.30, *p* < 0.05).

#### Cerebellar Gray Matter Analysis

Voxel-based morphometry (VBM) was used to identify differences in regional cerebellar volume between SPG7 and HS. The cerebellum was pre-processed individually using the Spatially Unbiased Infratentorial Template (SUIT) toolbox (48) implemented in Statistical Parametric Mapping version 8 [Wellcome Department of Imaging Neuroscience; SPM-8 (http://www.fil.ion.ucl.ac.uk/spm/)]. The procedure involved is as follows: cropping and isolating the cerebellum from the T1 anatomical images, normalizing each cropped image into SUIT space, and reslicing the probabilistic cerebellar atlas into individual subject spaces using the deformation parameters from normalization. The modulated gray matter (GM) probability maps were finally smoothed using an 8-mm FWHM Gaussian kernel, and statistical analyses were performed on the resulting GM maps with a voxel-wise two-sample t-test analysis for assessments of between-group differences in regional GM cerebellar volumes. The results were considered significant at *P* < 0.05 after FWE cluster-level correction (clusters formed with *P* < 0.005 at the uncorrected level).

#### Whole-Brain Gray Matter Analysis

To control for the effect of the accompanying cortical atrophy in SPG7 patients, whole-brain VBM was also performed. MDEFT T1 volumes were segmented into gray GM maps and registered to the Montreal Neurological Institute (MNI) space by means of the “New Segment” and “DARTEL” routines in SPM8 (http://www.fil.ion.ucl.ac.uk/spm/, Wellcome Trust Center for Neuroimaging, Institute of Neurology, University College London, UK) (49). VBM statistical analysis was performed to compare the GM maps between the patients and HS, entered as independent groups. The analysis excluded voxels in the cerebellum and was restricted to the cerebrum, which was entered as an explicit mask. For this analysis, intracranial volume was set as a covariate of no interest. T-contrasts were evaluated with voxel significance set at *p* < 0,001 and corrected for family-wise error (FWE) at the cluster level with the significance level set at *p* < 0.05.

#### Resting-State fMRI Data Pre-processing

fMRI data were pre-processed using SPM8 (http://www.fil.ion.ucl.ac.uk/spm/) and in-house software implemented in Matlab (The Mathworks Inc., Natick, MA, USA). For each subject, the first four volumes of the fMRI series were discarded to allow for T1 equilibration effects. The pre-processing steps included correction for head motion, compensation for slice-dependent time shifts, normalization to the EPI template in MNI coordinates provided with SPM8 and smoothing with a 3D Gaussian kernel with 8 mm^3^ full-width-at-half-maximum. For each data set, motion correction was checked to ensure that the maximum absolute shift did not exceed 2 mm and the maximum absolute rotation did not exceed 1.5°. Global temporal drift was removed using a third-order polynomial fit, the signal was regressed against the realignment parameters, and the signal was averaged over whole brain voxels to remove other potential sources of bias. All images were then filtered by a phase-insensitive band-pass filter (pass band 0.01–0.08 Hz) to reduce the effect of low-frequency drift and high-frequency physiological noise. Considering that GM atrophy may affect FC (50), every participant's total GM volume (absolute value) was also calculated and set as a nuisance variable for this analysis.

#### Definition of Regions of Interest and Seed-Based Analyses

The cerebellar DNs were chosen as regions of interest (ROIs) to test cerebello-cerebral FC differences in patients compared to controls. The choice of the DNs as ROIs was linked both to their role as the major cerebellar output channels to the cerebral cortex and to the DN alteration on MRI T2 sequences described in SPG7 patients (7). Left and right DN masks were separately extracted according to the SUIT atlas (48) and resliced in EPI standard space. A first-level SPM model was used to estimate the correlation between each voxel in the brain and the seed regions. The mean time course within each seed region of interest was extracted for every participant and used as a regressor in a first-level SPM analysis. The resulting beta images are thus equivalent to the Fisher z-transformed maps of the correlation coefficient. These images were taken to the second level for group analysis. At the second level, the group-level main effect of seed-to-voxel connectivity was tested by means of the F-test of significance for both left and right DNs. A two-sample *t*-test model was then used to explore differences in connectivity between patients and controls in each ROI. Between-group statistical significance was set at *p* < 0.05, FWE-corrected at the cluster level (clusters formed with uncorrected voxels; *p* < 0.005 at the voxel level).

## Results

### Behavioral Assessments

The scores of the performances obtained by SPG7 patients in the neuropsychological evaluation are reported in Supplementary Table 1. The assessments revealed the presence of CCAS according to the pathological scores obtained in at least three out of 10 subtests of the SSS. Moreover, the SPG7 group returned pathological results in specific verbal, language, and executive tasks of the neuropsychological assessment; specifically, 66% of SPG7 patients (four subjects) failed in both immediate and delayed recall of prose memory, naming objects described by the examiner, and phonological fluency; moreover, 83% of patients (five subjects) had problems in the naming verbs task and a poor time score in the Stroop task. Additionally, three patients had problems in the naming objects task, the long-term Rey's 15 words test, and the Rey-Osterrieth Figure (copy condition) (see Supplementary Table 1).

Regarding social cognition evaluation, the SPG7 group return pathological results in the ToM (66% of SPG7 patients), the RME total scores (83% of patients), and the “embarrassed” emotion of the EA test (100% of patients), while no differences compared to normative data were observed in the other emotions (see Supplementary Table 2).

### MRI Analysis

No subject was excluded due to motion artifacts in the MRI scans.

### Cerebellar VBM

**Figure 1:**
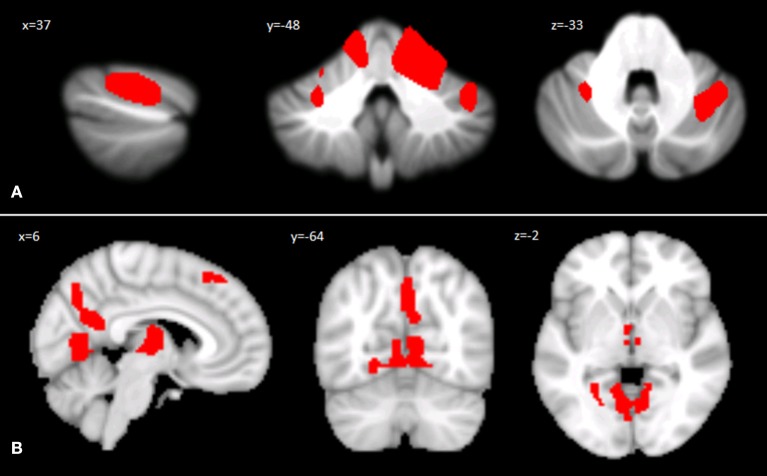
**(A)** Between-group voxel-based comparison of cerebellar GM volumes. Cerebellar regions showing patterns of significantly reduced GM in SPG7 compared to HS are reported and superimposed on sagittal (*x* = 37), coronal (*y* = −48), and axial (*z* = −33) sections of the Spatially Unbiased Infratentorial Template (SUIT; 41). Statistical significance was found at the cluster level (FWE = 0.05; cluster size: 31665), with peak voxels centered in the right lobule V and bilateral lobule VI with extension in the right Crus I. Images are shown according to neurological conventions. **(B)** Between-group voxel-based comparison of cerebral GM volumes. Cerebral regions showing patterns of significantly reduced GM compared to HS in SPG7 patients are reported in sagittal (*x* = 6), coronal (*y* = −64), and axial (*z* = −2) coordinates in Montreal Neurological Institute space. Left and right hemispheres are according to neurological conventions. See Tables 2A,B for detailed statistics.

Voxel-wise analysis of the cerebellar GM, comparing the SPG7 patients vs. HS, revealed a specific pattern of GM atrophy in the cerebellar cortex of SPG7 patients; indeed, VBM showed a large cluster of significantly decreased GM volume involving the right lobule V and bilateral lobule VI with extension in the right Crus I. No regions of increased cerebellar GM volume compared to HS were found in SPG7 patients. Detailed statistics and peak voxels showing the greatest significant difference in the cluster are reported in Table 2A. The results of cerebellar VBM are shown in Figure 1A.

**Table 2:** Statistics of cerebellar **(A)** and cerebral **(B)** GM differences (SPG7 < HS).

	**Cluster size (NoV)**	**Coordinates** ***x y z***			**Cluster peak *Z*-score**	**Cerebellar regions**
**(A)**	31665	16	−48	−11	3.84	R-Lobule V
		21	−40	−17	3.69	R-Lobule VI
		−23	−58	−14	3.62	L-Lobule VI
**Cerebral regions**						
**(B)**	1229	9	−63	−6	5.15	R-Lingual Gyrus
		−21	−64	−9	4.96	L-Lingual Gyrus
		−8	−66	−6	4.91	
	603	2	−67	42	4.66	R-Precuneus
		6	−55	22	4.20	
	760	9	−15	15	4.65	R-Thalamus
		−9	−18	12	4.13	L-Thalamus
		−6	−21	1	3.89	
	588	−4	29	57	4.18	L-Superior Frontal Gyrus
		−4	44	39	3.87	
		−15	30	57	3.65	

*MNI coordinates (x, y, z) in Montreal Neurological Institute Space and peak Z-score of the peak voxels showing greatest statistical differences in the clusters are reported. Only regions that survived after correction for multiple comparisons (FWE corrected p < 0.05) have been considered. NoV, number of voxels; L, left; R, right*.

### Cerebral VBM

Voxel-wise analysis of the cerebral GM maps revealed a significant pattern of GM atrophy in the cerebral cortex of SPG7 patients compared to controls. More specifically, the VBM analysis showed different clusters of significantly decreased GM volume involving cortical and subcortical regions in the left and right cerebral hemispheres, such as the bilateral lingual gyrus, the bilateral thalamus, the right precuneus and cingulate gyrus, and the left superior frontal gyrus and frontal pole. Detailed statistics and peak voxels showing the greatest significant differences in the clusters are reported in Table 2B. The results of cerebral voxel-based morphometry are shown in Figure 1B.

### Seed-Based Analysis of Dentate-Cerebral Functional Connectivity

**Figure 2:**
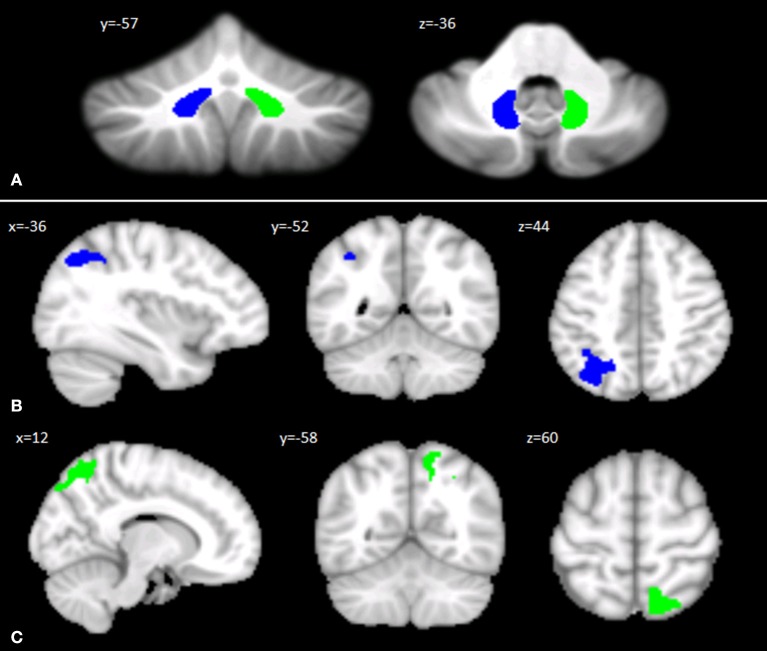
**(A)** Seed regions in the cerebellar dentate nuclei. Coronal (*y*) and axial (*z*) views of the generated left (blue) and right (green) dentate nuclei used as regions of interest for assessing cerebello-cerebral FC. The left and right cerebellar DNs are superimposed on the Spatially Unbiased Infratentorial Template of the cerebellum and brainstem (SUIT, 41). **(B,C)** Patterns of DN functional connectivity with the cerebral cortex. Patterns of increased dentate–cerebral FC are shown for the left **(B)** and right **(C)** DN in different colors (blue and green, respectively). Sagittal (*x*), coronal (*y*), and axial slices (*z*) in Montreal Neurological Institute space. Clusters of increased dentate-cerebral FC were considered significant after correction for multiple comparisons (FWE corrected *p* < 0.05). Images are shown according to neurological conventions. See Supplementary Table 3 for detailed statistics.

The left and right DN masks used as ROIs for the FC analysis are shown in Figure 2A. The seed-based analysis revealed significant FC alterations in SPG7 patients vs. HS: specifically, a significant cluster of increased FC was found between the left DN and ipsilateral cerebral regions, including the lateral occipital cortex, the supramarginal gyrus, and the superior parietal lobule (Figure 2B). Similarly, a significant cluster of increased FC was found between the right DN and ipsilateral cerebral regions, including the lateral occipital cortex and the precuneus (Figure 2C). No regions of significantly decreased FC with the left and right DNs were found. Detailed statistics and peak voxels showing the greatest significant difference in the clusters are reported in Supplementary Table 3.

## Discussion

The present preliminary study provides the first evidence of specific cerebellar modifications that affect long-distance regions of the brain in a cohort of SPG7 patients, showing a link between the anatomical alterations and the clinical symptoms found in terms of cognitive and social ability impairments.

Our cohort of SPG7 patients, all of whom showed signs of cerebellar atrophy on the diagnostic brain MRI, was assessed by means of high-resolution brain MRI techniques, in parallel with neuropsychological and social tasks for scoring the patients' cognitive and emotional performances.

In agreement with previous data obtained in patients with various cerebellar pathologies (11, 16, 51–56), the results of the neuropsychological assessments indicated the presence of CCAS and serious impairments in specific verbal, language, and executive tasks in our SPG7 cohort.

Additionally, the in-depth investigation of social skills indicated specific difficulties in the attribution of the “embarrassed” emotion, in the automatic attribution of relevant mental states regardless of the context (RME), and in theory of mind. Interestingly, a recent study by Clausi et al. (9) described a similar impairment in patients affected by cerebellar atrophy. Indeed, they were characterized by a lack of ability to “tune in” to the mental state of another person both at an unconscious and an automatic level, as assessed by RME, and at a more complex and conscious level, as assessed by ToM. In the same way, the low score specifically obtained in the “embarrassed” emotion is consistent with the high level of social interaction implied by this complex emotion (57). Altogether, our results not only confirm executive dysfunctions and impairment in facial emotional expressions, already described in a single SPG7 case (23, 58), but also allow us to delineate a multifaceted cognitive and social profile as part of the SPG7 complex phenotype.

Importantly, the neuroimaging studies suggested the association of characteristic structural brain alterations with such cognitive/emotional features, as cerebellar VBM showed GM reduction in the right lobule V and Crus I and the bilateral lobule VI. Interestingly, while the anterior lobule V is more involved in somatosensory and motor aspects, both the postero-lateral cerebellum lobule VI and Crus I are associated with the processing of cognitive and emotional information (21, 59), and Crus I is also involved in the mentalizing network of social abilities (60). Furthermore, cerebral VBM indicated GM reduction in the lingual gyrus, precuneus, thalamus, and superior frontal gyrus. It must be noted that the lingual gyrus plays a role in the identification and recognition of words, while the precuneus and superior frontal gyrus are involved in different aspects of social interactions and are included in the default mode network (DMN) (61–65).

Thus, in view of these data, the verbal memory and language impairment shown by our SPG7 patients might be related to the atrophy in the cerebellar posterior lobules and in the lingual gyrus, while the “theory of mind” alterations might be linked to the atrophy in the precuneus and superior frontal gyrus, supporting the relevance of the functional alteration of cerebello-cerebral networks implicated in different cognitive and social functions (10, 12, 66).

In this regard, our functional connectivity results support this hypothesis. Indeed, an abnormal pattern of FC was found between both the right and left DNs and specific cortical areas in our SPG7 patients, characterized by an increased FC of the right DN with ipsilateral regions of the lateral occipital cortex, known to be involved in somatosensory processing, and the precuneus, which is part of the DMN and is involved in social cognition ability. The left DN showed an increased FC with the ipsilateral occipital cortex, the left supramarginal gyrus, and the left superior parietal lobule. In particular, the supramarginal gyrus is involved not only in retrieval and episodic memory but also in social interaction, similar to the left superior parietal lobe (61–65), confirming the cerebello-cortical dysregulation in different networks involved in social functioning (9, 12, 61, 64).

We hypothesize that hyperconnectivity is the pathological manifestation of altered functional interaction between the DN and cerebral regions. This dysregulation is conceivable considering cerebellar cortical degeneration. Indeed, as a result of the degenerative process, the increased FC evidenced in the present study suggests a release of the inhibitory control that is normally exerted by the cerebellar cortex on the DNs. As a consequence, the DNs increase their excitatory outputs to the cerebral cortex, thus resulting in a pattern of overconnectivity that impairs the optimization of functions in the dentate-thalamo-cortical tract subserving cognitive and social functioning (12, 67).

## Conclusion

This study reported a preliminary characterization of the cognitive and emotional profile of the SPG7 complex phenotype, which might be explained by cerebellar and cortical structural alterations and the functional dysregulation of specific cerebello-cortical networks.

Moreover, in the current study, the cerebello-cortical overconnectivity results were strictly ipsilateral, in agreement with previous studies showing a similar altered connection pattern between the cerebellum and cerebral cortex (8, 11, 68, 69). In this regard, we emphasize that although the majority of cerebello-cerebral anatomical connections are known to be contralateral (70), functional connectivity can be independent from the underlying structural (anatomical) connectivity (71) since FC typically refers to the neural synchronization between separated brain regions (72).

Overall, it is important to note that the combined use of structural and functional connectivity investigations has been fundamental to characterizing, for the first time, the anatomical specificity of SPG7 pathology.

The major limitation of this study is the small sample of SPG7 patients, which is a product of the rarity of this neurogenetic condition, and the presence of only male subjects. Moreover, considering that the cluster-extent based threshold should be ideally set at 0.001 (73), another limitation to consider is that FC cerebellar VBM and FC analysis gave significant results only when using a more permissive *p* threshold (0,005).

In spite of these limitations, the present results give a preliminary profile of the general cognitive and social alterations associated with this pathology. Hopefully, these issues will be addressed in future studies including larger SPG7 cohorts.

## Data Availability Statement

The raw data supporting the conclusions of this article will be made available by the authors, without undue reservation, to any qualified researcher.

## Ethics Statement

The studies involving human participants were reviewed and approved by Ethics Committee of the IRCCS Fondazione Santa Lucia. The patients/participants provided their written informed consent to participate in this study.

## Author Contributions

ML drafting and revising the manuscript, study concept and design, and analysis and interpretation of data. Accepts responsibility for conducting the research and for final approval, acquisition of data, and study supervision. GO drafting and revising the manuscript, study concept, acquisition, and analysis of MRI data. SC and LS drafting and revising the manuscript and acquisition of data. VR revising the manuscript and acquisition of molecular diagnosis. MB supervision of MRI analysis and revising the manuscript. FS supervision of molecular diagnosis analysis and revising the manuscript. GS enrolment of patients, supervision of molecular diagnosis analysis, and revising the manuscript. ML drafting and revising the manuscript, study concept and design, analysis and interpretation of data, and accepts responsibility for conducting research and for final approval and study supervision.

### Conflict of Interest

The authors declare that the research was conducted in the absence of any commercial or financial relationships that could be construed as a potential conflict of interest.
