# Supplementary material for: Cerebello-Cortical Alterations Linked to Cognitive and Social Problems in Patients With Spastic Paraplegia Type 7: A Preliminary Study
Source: Front Neurol. 2020 Feb 25;11:82. doi: 10.3389/fneur.2020.00082 (PMC7053515; doi:10.3389/fneur.2020.00082)
Supplement: Supplementary file 1 [file Data_Sheet_1.doc]

**Supplementary Material**

Table 1. Neuropsychological profile of each SPG7 patient
CB1

|  | **Test** | **Total Raw score** | **N° of deficit items** | **Cut off  n° of items** |
| --- | --- | --- | --- | --- |
| ***Cerebellar Cognitive Affective Syndrome*** | Schmahmann Syndrome Scale | 55 | 8* | >2 |
| **Cognitive Domains** | **Tests** | **Raw score** | **Correct score** | **Cut off Normative data** |
| *INTELLECTUAL LEVEL* |  |  |  |  |
|  | WAIS-IV IQ: | 47 | 60* | <70 |
|  | Raven Progressive Matrices | 24 | 24,3 | 18,96 |
| *VERBAL MEMORY* |  |  |  |  |
|  | Rey’s 15 mots short term | 43 | 42,2 | 28,53 |
|  | Rey’s 15 mots long term  Prose Memory (immediate) | 10  6,6 | 9,6  6,5 | <4,69  <4.75 |
|  | Prose Memory (recall) | 5,3 | 5,3 | ≤4,5 |
|  | Forward digit span | 4* |  | 7+/-2 |
|  | Backward digit span | 2* |  | 5+/-2 |
| *VISUOSPATIAL MEMORY* |  |  |  |  |
|  | Rey-Osterrieth figure (recall) | 11,5 | 9,5 | 9,46≤ |
|  | Forward Corsi | 5 | 5 | 7+/-2 |
|  | Backward Corsi | 4 |  | 5+/-2 |
| *VISUOSPATIAL ABILITY* |  |  |  |  |
|  | Rey-Osterrieth figure (copy) | 17 | 17,8* | ≤28,87 |
| *LANGUAGE* |  |  |  |  |
|  | Naming objects | 27* |  | 28< |
|  | Naming verbs | 21* |  | 26< |
|  | Naming objects described by the examiner | 15 |  | 14< |
|  | Generation of sentences | 8 | 7 | ≤6,25 |
|  | Token Test | n.e. | n.e. | <32 |
| *EXECUTIVE FUNCTIONS* |  |  |  |  |
|  | Phonological fluency | 15 | 16,5* | 17,35 |
|  | Verbal fluency | 3 | ≤ 2,5°perc* | <10°perc |
|  | Wisconsin Card Sorting Test: |  |  |  |
|  | Total Number of errors | 77 | 62 PS* | <85 PS |
|  | Total Number of perseverative errors | 48 | 62 PS* | <85 PS |
|  | Tower of London | n.e. | n.e. | 28-35 |
|  | Stroop Test: |  |  |  |
|  | Time score | 50,69 | 50,19* | ≥36,92 |
|  | Errors score | 1 | 0,75 | ≥4,24 |
| *ATTENTION* |  |  |  |  |
|  | Multiple features targets cancellation task- Accuracy |  | 1 | <0,869 |
|  | Trail Making Test –Time: |  |  |  |
|  | Part A | 110s | 97* | >93s |
|  | Part B | 277s | 229 | >282s |
|  | Part B-A | 167 | 133 | >186s |

Perc= percentile; PS= standard point; s= seconds; n.e.= not evaluable. ***** value under the cut-off normative data

CB2

|  | **Test** | **Total Raw score** | **N° of deficit items** | | **Cut off n° of items** |
| --- | --- | --- | --- | --- | --- |
| ***Cerebellar Cognitive Affective Syndrome*** | Schmahmann Syndrome Scale | 67 | 7* | | >2 |
| **Cognitive Domains** | **Tests** | **Raw score** | **Correct score** | | **Cut off Normative data** |
| *INTELLECTUAL LEVEL* |  |  |  | |  |
|  | WAIS-IV IQ: | 78 | 83 | | <70 |
|  | Raven Progressive Matrices | 35 | 32,8 | | 18,96 |
| *VERBAL MEMORY* |  |  |  | |  |
|  | Rey’s 15 mots short term | 56 | 52,2 | | 28,53 |
|  | Rey’s 15 mots long term  Prose Memory (immediate) | 13  2,2 | 12  2,1* | | <4,69  <4,75 |
|  | Prose Memory (recall) | 2,1 | 1,8* | | ≤4,5 |
|  | Forward digit span | 5 |  | | 7+/-2 |
|  | Backward digit span | 4 |  | | 5+/-2 |
| *VISUOSPATIAL MEMORY* |  |  |  | |  |
|  | Rey-Osterrieth figure (recall) | 14,5 | 11,1 | | 9,46≤ |
|  | Forward Corsi | 4 | 3,75 | | 7+/-2 |
|  | Backward Corsi | 6 |  | | 5+/-2 |
| *VISUOSPATIAL ABILITY* |  |  |  | |  |
|  | Rey-Osterrieth figure (copy) | 23 | 22* | | ≤28,87 |
| *LANGUAGE* |  |  |  | |  |
|  | Naming objects | 29 |  | | 28< |
|  | Naming verbs | 23* |  | | 26< |
|  | Naming objects described by the examiner | 12* |  | | 14< |
|  | Generation of sentences | 14 | 11,5 | | ≤6,25 |
|  | Token Test | n.e. | n.e. | | <32 |
| *EXECUTIVE FUNCTIONS* |  |  |  | |  |
|  | Phonological fluency | 22 | | 17,3* | 17,35 |
|  | Verbal fluency | 13 | | > 10° - 25°perc < | <10°perc |
|  | Wisconsin Card Sorting Test: |  | |  |  |
|  | Total Number of errors | 20 | | 108 PS | <85 PS |
|  | Total Number of perseverative errors | 9 | | 119 PS | <85 PS |
|  | Tower of London | 30 | |  | 27-36 |
|  | Stroop Test: |  | |  |  |
|  | Time score | 17,5 | | 19 | ≥36,92 |
|  | Errors score | 0 | | 0,25 | ≥4,24 |
| *ATTENTION* |  |  | |  |  |
|  | Multiple features targets cancellation task- Accuracy |  | | 1 | <0,869 |
|  | Trail Making Test –Time: |  | |  |  |
|  | Part A | 79s | | 73 | >93s |
|  | Part B | 120s | | 104 | >282s |
|  | Part B-A | 41 | | 31 | >186s |

Perc= percentile; PS= standard point; s= seconds; n.e.= not evaluable. ***** value under the cut-off normative data

CB3

|  | **Test** | **Total Raw score** | **N° of deficit items** | **Cut off n° of items** |
| --- | --- | --- | --- | --- |
| ***Cerebellar Cognitive Affective Syndrome*** | Schmahmann Syndrome Scale | 67 | 6* | >2 |
| **Cognitive Domains** | **Tests** | **Raw score** | **Correct score** | **Cut off Normative data** |
| *INTELLECTUAL LEVEL* |  |  |  |  |
|  | WAIS-IV IQ: | 76 | 82 | <70 |
|  | Raven Progressive Matrices | 30 | 28,3 | 18,96 |
| *VERBAL MEMORY* |  |  |  |  |
|  | Rey’s 15 mots short term | 34 | 32 | 28,53 |
|  | Rey’s 15 mots long term  Prose Memory (immediate) | 5  2 | 4,5*  2* | <4,69  <4,75 |
|  | Prose Memory (recall) | 3,1 | 2,9* | ≤4,5 |
|  | Forward digit span | 5 |  | 7+/-2 |
|  | Backward digit span | 3 |  | 5+/-2 |
| *VISUOSPATIAL MEMORY* |  |  |  |  |
|  | Rey-Osterrieth figure (recall) | 12,5 | 10,1 | 9,46≤ |
|  | Forward Corsi | 5 | 4,75 | 7+/-2 |
|  | Backward Corsi | 4 |  | 5+/-2 |
| *VISUOSPATIAL ABILITY* |  |  |  |  |
|  | Rey-Osterrieth figure (copy) | 32 | 31,3 | ≤28,87 |
| *LANGUAGE* |  |  |  |  |
|  | Naming objects | 23* |  | 28< |
|  | Naming verbs | 23* |  | 26< |
|  | Naming objects described by the examiner | 13* |  | 14< |
|  | Generation of sentences | 0 | 0* | ≤6,25 |
|  | Token Test | n.e. | n.e. | <32 |
| *EXECUTIVE FUNCTIONS* |  |  |  |  |
|  | Phonological fluency | 20 | 15,8* | 17,35 |
|  | Verbal fluency | 13 | > 10° - 25°perc < | <10°perc |
|  | Wisconsin Card Sorting Test: |  |  |  |
|  | Total Number of errors | 45 | 81* PS | <85 PS |
|  | Total Number of perseverative errors | 24 | 92 PS | <85 PS |
|  | Tower of London | 29 |  | 27-36 |
|  | Stroop Test: |  |  |  |
|  | Time score | 53,43 | 52,67* | ≥36,92 |
|  | Errors score | 2 | 2 | ≥4,24 |
| *ATTENTION* |  |  |  |  |
|  | Multiple features targets cancellation task- Accuracy |  | 0,77* | <0,869 |
|  | Trail Making Test –Time: |  |  |  |
|  | Part A | 91s | 85 | >93s |
|  | Part B | 114s | 98 | >282s |
|  | Part B-A | 23 | 13 | >186s |

Perc= percentile; PS= standard point; s= seconds; n.e.= not evaluable. ***** value under the cut-off normative data

CB4

|  | **Test** | **Total Raw score** | **N° of deficit items** | **Cut off n° of items** |
| --- | --- | --- | --- | --- |
| ***Cerebellar Cognitive Affective Syndrome*** | Schmahmann Syndrome Scale | 101 | 3* | >2 |
| **Cognitive Domains** | **Tests** | **Raw score** | **Correct score** | **Cut off Normative data** |
| *INTELLECTUAL LEVEL* |  |  |  |  |
|  | WAIS-IV IQ: | 82 | 86 | <70 |
|  | Raven Progressive Matrices | 33 | 29,1 | 18,96 |
| *VERBAL MEMORY* |  |  |  |  |
|  | Rey’s 15 mots short term | 31 | 25,7* | 28,53 |
|  | Rey’s 15 mots long term  Prose Memory (immediate) | 6  0 | 4,5*  0* | <4,69  <4,75 |
|  | Prose Memory (recall) | 0 | 0* | ≤4,5 |
|  | Forward digit span | 7 |  | 7+/-2 |
|  | Backward digit span | 3 |  | 5+/-2 |
| *VISUOSPATIAL MEMORY* |  |  |  |  |
|  | Rey-Osterrieth figure (recall) | 14 | 9,7 | 9,46≤ |
|  | Forward Corsi | 5 | 3,5 | 7+/-2 |
|  | Backward Corsi | 4 |  | 5+/-2 |
| *VISUOSPATIAL ABILITY* |  |  |  |  |
|  | Rey-Osterrieth figure (copy) | 24 | 21,7* | ≤28,87 |
| *LANGUAGE* |  |  |  |  |
|  | Naming objects | 30 |  | 28< |
|  | Naming verbs | 25* |  | 26< |
|  | Naming objects described by the examiner | 16* |  | 14< |
|  | Generation of sentences | 13 | 9,75 | ≤6,25 |
|  | Token Test | 36 | 33 | <32 |
| *EXECUTIVE FUNCTIONS* |  |  |  |  |
|  | Phonological fluency | 31 | 22,2 | 17,35 |
|  | Verbal fluency | 16 | 25°perc | <10°perc |
|  | Wisconsin Card Sorting Test: |  |  |  |
|  | Total Number of errors | 19 | 100 PS | <85 PS |
|  | Total Number of perseverative errors | 9 | 100 PS | <85 PS |
|  | Tower of London | 30 |  | 27-36 |
|  | Stroop Test: |  |  |  |
|  | Time score | 19,97 | 23,47* | ≥36,92 |
|  | Errors score | 0 | 0,75 | ≥4,24 |
| *ATTENTION* |  |  |  |  |
|  | Multiple features targets cancellation task- Accuracy |  | 0,88 | <0,869 |
|  | Trail Making Test –Time: |  |  |  |
|  | Part A | 78s | 78 | >93s |
|  | Part B | 158s | 167 | >282s |
|  | Part B-A | 80 | 89 | >186s |

Perc= percentile; PS= standard point; s= seconds. ***** value under the cut-off normative data

CB5

|  | **Test** | **Total Raw score** | **N° of deficit items** | **Cut off n° of items** |
| --- | --- | --- | --- | --- |
| ***Cerebellar Cognitive Affective Syndrome*** | Schmahmann Syndrome Scale | 67 | 6* | >2 |
| **Cognitive Domains** | **Tests** | **Raw score** | **Correct score** | **Cut off Normative data** |
| *INTELLECTUAL LEVEL* |  |  |  |  |
|  | WAIS-IV IQ: | 60 | 70 | <70 |
|  | Raven Progressive Matrices | 30 | 25,2 | 18,96 |
| *VERBAL MEMORY* |  |  |  |  |
|  | Rey’s 15 mots short term | 33 | 22,1* | 28,53 |
|  | Rey’s 15 mots long term  Prose Memory (immediate) | 7  0 | 3,5*  0* | <4,69  <4,75 |
|  | Prose Memory (recall) | 0 | 0* | ≤4,5 |
|  | Forward digit span | 6 |  | 7+/-2 |
|  | Backward digit span | 4 |  | 5+/-2 |
| *VISUOSPATIAL MEMORY* |  |  |  |  |
|  | Rey-Osterrieth figure (recall) | 9 | 1,4* | 9,46≤ |
|  | Forward Corsi | 6 | 5,25 | 7+/-2 |
|  | Backward Corsi | 6 |  | 5+/-2 |
| *VISUOSPATIAL ABILITY* |  |  |  |  |
|  | Rey-Osterrieth figure (copy) | 27 | 24,4* | ≤28,87 |
| *LANGUAGE* |  |  |  |  |
|  | Naming objects | 22* |  | 28< |
|  | Naming verbs | 22* |  | 26< |
|  | Naming objects described by the examiner | 12* |  | 14< |
|  | Generation of sentences | n.e. | n.e. | ≤6,25 |
|  | Token Test | 32 | 30* | <32 |
| *EXECUTIVE FUNCTIONS* |  |  |  |  |
|  | Phonological fluency | 14 | 7,2* | 17,35 |
|  | Verbal fluency | 10 | 10°perc* | <10°perc |
|  | Wisconsin Card Sorting Test: |  |  |  |
|  | Total Number of errors | 8 | 138 PS | <85 PS |
|  | Total Number of perseverative errors | 5 | 138 PS | <85 PS |
|  | Tower of London | 33 |  | 27-36 |
|  | Stroop Test: |  |  |  |
|  | Time score | 13,42 | 29,17* | ≥36,92 |
|  | Errors score | 0 | 2 | ≥4,24 |
| *ATTENTION* |  |  |  |  |
|  | Multiple features targets cancellation task- Accuracy |  | 0,84* | <0,869 |
|  | Trail Making Test –Time: |  |  |  |
|  | Part A | 42,56s | 55,56 | >93s |
|  | Part B | 130,65s | 175,65 | >282s |
|  | Part B-A | 88,09 | 119,09 | >186s |

Perc= percentile; PS= standard point; s= seconds; n.e.= not evaluable. ***** value under the cut-off normative data

CB6

|  | **Test** | **Total Raw score** | **N° of deficit items** | **Cut off n° of items** |
| --- | --- | --- | --- | --- |
| ***Cerebellar Cognitive Affective Syndrome*** | Schmahmann Syndrome Scale | 103 | 2 | >2 |
| **Cognitive Domains** | **Tests** | **Raw score** | **Correct score** | **Cut off Normative data** |
| *INTELLECTUAL LEVEL* |  |  |  |  |
|  | WAIS-IV IQ : | 101 | 101 | <70 |
|  | Raven Progressive Matrices | 35 | 29,7 | 18,96 |
| *VERBAL MEMORY* |  |  |  |  |
|  | Rey’s 15 mots short term | 56 | 46,7 | 28,53 |
|  | Rey’s 15 mots long term  Prose Memory (immediate) | 12  6,6 | 9,2  6,2 | <4,69  <4,75 |
|  | Prose Memory (recall) | 5,3 | 4,5 | ≤4,5 |
|  | Forward digit span | 6 |  | 7+/-2 |
|  | Backward digit span | 5 |  | 5+/-2 |
| *VISUOSPATIAL MEMORY* |  |  |  |  |
|  | Rey-Osterrieth figure (recall) | 26 | 19,3 | 9,46≤ |
|  | Forward Corsi | 5 | 4,25 | 7+/-2 |
|  | Backward Corsi | 7 |  | 5+/-2 |
| *VISUOSPATIAL ABILITY* |  |  |  |  |
|  | Rey-Osterrieth figure (copy) | 36 | 32,9 | ≤28,87 |
| *LANGUAGE* |  |  |  |  |
|  | Naming objects | 29 |  | 28< |
|  | Naming verbs | 26 |  | 26< |
|  | Naming objects described by the examiner | 16 |  | 14< |
|  | Generation of sentences | n.e. | n.e. | ≤6,25 |
|  | Token Test | 35 | 32 | <32 |
| *EXECUTIVE FUNCTIONS* |  |  |  |  |
|  | Phonological fluency | 37 | 27 | 17,35 |
|  | Verbal fluency | 25 | > 90° - 75°perc < | <10°perc |
|  | Wisconsin Card Sorting Test: |  |  |  |
|  | Total Number of errors | 24 | 92 PS | <85 PS |
|  | Total Number of perseverative errors | 9 | 100 PS | <85 PS |
|  | Tower of London | 33 |  | 27-36 |
|  | Stroop Test: |  |  |  |
|  | Time score | 28,05 | 38,55* | ≥36,92 |
|  | Errors score | 0 | 1,5 | ≥4,24 |
| *ATTENTION* |  |  |  |  |
|  | Multiple features targets cancellation task- Accuracy |  | 0,92 | <0,869 |
|  | Trail Making Test –Time: |  |  |  |
|  | Part A | 37,34s | 47,34 | >93s |
|  | Part B | 76,59s | 115,59 | >282s |
|  | Part B-A | 39,25 | 69,25 | >186s |

Perc= percentile; PS= standard point; s= seconds; n.e.= not evaluable. ***** value under the cut-off normative data

Table 2. Social Cognition profile of SPG7

|  | | **EA** | | | | | | | **RME** | **ToM** |
| --- | --- | --- | --- | --- | --- | --- | --- | --- | --- | --- |
| **Subjects** | **age** | **sadness** | **fear** | **embarrassment** | **disgust** | **happiness** | **anger** | **jealousy** |
| CB1 | 54 | 7 | 9 | 5* | 2 | 10 | 1* | 0* | 10* | 8* |
| CB2 | 53 | 5* | 9 | 0* | 3 | 9* | 6 | 2 | 19* | 10* |
| CB3 | 55 | 7 | 7* | 7* | 1* | 9* | 7 | 1 | 15* | 9* |
| CB4 | 54 | 6 | 8 | 7* | 1* | 8* | 1* | 2 | 26 | 12 |
| CB5 | 23 | 8 | 7* | 4* | 3 | 10 | 6 | 0* | 12* | 9* |
| CB6 | 39 | 9 | 9 | 7* | 2 | 10 | 6 | 3 | 23* | 12 |
| **Cut off** | | **6<** | **8<** | **8<** | **2<** | **10<** | **6<** | **1<** | **age 18-40 : 25,0<**  **age 41-60 : 23,1<** | **12<** |

* value under the cut-off normative data
EA= emotional attribution test; RME= reading the mind in the eyes test; ToM= Theory of Mind test

Table 3. Statistics of left (L-) and right (R-) DN functional connectivity results (SPG7>HS)

|  |  |  | | |  |  |
| --- | --- | --- | --- | --- | --- | --- |
|  | **Cluster Size**  **(NoV)** | **Coordinates** | | | **Cluster Peak *Z*-score** | **Cerebral regions** |
|  |  | **x** | **y** | **z** |  |  |
| **L-DN** | **442** | -28 | -64 | 40 | **3.94** | L-Lateral Occipital Cortex |
|  |  | -32 | -72 | 42 | **3.92** | L-Lateral Occipital Cortex |
|  |  | -36 | -50 | 44 | **2.79** | L-Supramarginal Gyrus |
|  |  |  |  |  |  |  |
| **R-DN** | **425** | 14 | -66 | 62 | **7.81** | R-Lateral Occipital Cortex |
|  |  | 12 | -58 | 60 | **6.71** | R-Precuneus |
|  |  | 12 | -72 | 52 | **6.59** | R-Lateral Occipital Cortex |
|  | | | | | | |

MNI coordinates (x, y, z) in the Montreal Neurological Institute space and peak Z-score of the peak voxels showing greatest statistical differences in the clusters are reported. Only regions that survived after correction for multiple comparisons (FWE corrected p <0.05) have been considered. NoV= number of voxels; L: left; R: right.

*Appendix*

Social cognition tasks description:

The Reading the Mind in the Eyes test (RME) (Baron-Cohen et al., 2001; Serafin and Surian, 2004) was used to assess the first stage (automatic) of attribution of relevant mental state to others regardless the context. Indeed, within the face, the eyes are the most important contact between agents (Hainline, 1978; Maurer, 1985). This test is made up of 36 photos of actors’ eyes and for each, the participants have to choose from four alternative words the one that best described what the person in the photograph is thinking or feeling. Responses were scored 1 or 0 for correctness. The participants have to put themselves into the mind of the person shown in the photo and ‘tune in’ to their relevant mental state. The core of the RME involves the matching of the semantic definition of a mental state (e.g. worried, annoyed) to the picture of the eye-region expression. This is assumed to involve an unconscious, automatic and rapid matching of the past memories/categorization concerning similar expressions with lexicon of mental state terms to arrive at a judgement of which word the eyes most closely match (Baron-Cohen et al., 2001). Automaticity and categorization are important determinants of the meaning of gesture, speech, and expression (Knutson et al., 2007) and are fundamental to the prediction of the others mental state.

The Emotion Attribution test (EA) (Blair and Cipollotti, 2000; Prior et al., 2003) was used to assess the ability to attribute emotions to others in a social context. In this test, 58 short stories describing an emotional situation were presented to the subject and required to provide a one-word description of how the main character might feel in that situation. The sentences were designed to elicit sadness, fear, embarrassment, disgust, happiness, anger or envy. The sequential events of the story are explicit and univocal, requiring a low level of prediction about the emotional consequences of the. The correct answer is based on the coherent expectations on the social interaction.

The Theory of Mind (Happé, 1994; Blair and Cipollotti, 2000; Van Harskamp et al 2005; Prior et al., 2003) was used to assess the more advanced concepts of the ToM, such as double bluff, white lies, and persuasion. Participant was presented with 13 stories describing naturalistic social situations and was asked to interpret and justify the behaviour of the main character. The subject has to identify accurately the underlying intention behind a character’s utterance that was not literally true and to explain why the main character acted in a particular manner. Successful performance requires attribution of mental states such as desires, beliefs or intentions, and higher order mental states such as one character’s belief about what another character knows. The sequential events of the story are not univocal and, requiring a high level of prediction about the consequence of the events. The correct answer is based on the capacity to make a choice taking into account different expectations about the social interaction.
